# SaliVISION: a rapid saliva-based COVID-19 screening and diagnostic test with high sensitivity and specificity

**DOI:** 10.1038/s41598-022-09718-4

**Published:** 2022-04-06

**Authors:** Samuel M. DeFina, Jianhui Wang, Lei Yang, Han Zhou, Jennifer Adams, William Cushing, Beth Tuohy, Pei Hui, Chen Liu, Kien Pham

**Affiliations:** 1 Department of Pathology, Yale School of Medicine, Yale University, New Haven, CT USA; 2 Department of Laboratory Medicine, Yale School of Medicine, Yale University, New Haven, CT USA; 3 Department of Internal Medicine, Yale School of Medicine, Yale University, New Haven, CT USA; 4 Yale New Haven Hospital, New Haven, CT USA; 5 Yale University Health Services, Yale University, New Haven, CT USA

**Keywords:** Pathology, Reverse transcription polymerase chain reaction

## Abstract

The Coronavirus disease 2019 (COVID-19) pandemic-caused by the severe acute respiratory syndrome coronavirus 2 (SARS-CoV-2)– has posed a global threat and presented with it a multitude of economic and public-health challenges. Establishing a reliable means of readily available, rapid diagnostic testing is of paramount importance in halting the spread of COVID-19, as governments continue to ease lockdown restrictions. The current standard for laboratory testing utilizes reverse transcription quantitative polymerase chain reaction (RT-qPCR); however, this method presents clear limitations in requiring a longer run-time as well as reduced on-site testing capability. Therefore, we investigated the feasibility of a reverse transcription looped-mediated isothermal amplification (RT-LAMP)-based model of rapid COVID-19 diagnostic testing which allows for less invasive sample collection, named SaliVISION. This novel, two-step, RT-LAMP assay utilizes a customized multiplex primer set specifically targeting SARS-CoV-2 and a visual report system that is ready to interpret within 40 min from the start of sample processing and does not require a BSL-2 level testing environment or special laboratory equipment. When compared to the SalivaDirect and Thermo Fisher Scientific TaqPath RT-qPCR testing platforms, the respective sensitivities of the SaliVISION assay are 94.29% and 98.28% while assay specificity was 100% when compared to either testing platform. Our data illustrate a robust, rapid diagnostic assay in our novel RT-LAMP test design, with potential for greater testing throughput than is currently available through laboratory testing and increased on-site testing capability.

## Introduction

The Coronavirus disease 2019 (COVID-19) pandemic, caused by the severe acute respiratory syndrome coronavirus 2 (SARS-CoV-2)^1^, has had an immense global impact through a variety of media-e.g., economic, psychological, public health, etc^2^. In the United states, alone, there have been 29.8 million reported COVID-19 cases, and over 540,000 reported deaths^3^. Further, while it is known that people with pre-existing conditions are at greater risk for COVID-19 related complications, we have yet to identify all high-risk populations. Moreover, a recent systematic review of several studies, highlighted varying rates of asymptomatic spreaders across different populations, regularly projecting asymptomatic spreaders to account for 50% of all COVID-19 cases^4^, underscoring the need for additional methods of containment for COVID-19^5–9^. Additionally, among symptomatic populations, it has been reported that people are most contagious in the first 5 days after symptom onset, typically before presentation of severe symptoms, further compounding the level of complexity involved in early detection of COVID-19^4,10,11^. This suggests that increased social distancing and mask-wearing efforts, alone, are not sufficient in curbing the spread of COVID-19, but additionally, the early detection and quarantine of asymptomatic persons. Furthermore, despite recent vaccination efforts in conjunction with stringent social distancing practices, large-scale, readily accessible rapid testing will be of paramount importance in slowing the spread of COVID-19 and returning to some resemblance of pre-pandemic life^12–15^.

Current COVID-19 testing efforts rely upon sample collection of saliva and more invasive, nasopharyngeal (NP) swabbing for conventional methods for viral detection, such as reverse transcription quantitative polymerase chain reaction (RT-qPCR)^16–18^. As it stands, the employment of saliva collection as a means of sample collection bears marked advantages, compared to NP swab collection, especially with regards to method tolerability. Particularly, NP swabbing performed by healthcare workers has garnered a sense of testing-apprehension among the general population due to the unpleasantness of this modality. On the other hand, saliva collection can be performed using several different methods, thereby increasing its practicality among demographics who might otherwise opt to forego testing. Moreover, the SARS-CoV-2 virus is capable of replicating within cells that line the oral mucosa at a level that allows for detection, even among people who are asymptomatic, and does so for up to 20 days, underscoring the robustness of saliva as source for testing^10,19,20^. In addition to the abundance of current suboptimal test collection methods, the utilization of laboratory RT-qPCR requires an expensive, stationary, collection of laboratory equipment, in addition to extended sample-processing times^21,22^. Typically, diagnostic laboratory testing utilizing RT-qPCR requires several processing steps, with unique reagents for each step, which often result in sample-processing times that exceed several hours. Consequently, as testing demands increase, so to do turn-around-times for test results, which when combined with reagent/supply shortages for the necessary equipment, clinical-laboratory diagnostic testing throughput is severely hindered. Therefore, development of rapid viral-genome amplification methods has become the focus of testing efforts due to increased throughput, point-of-care (POC) capability^23–25^, and potential for early detection of asymptomatic carriers of COVID-19.

Loop-mediated isothermal amplification (LAMP) is method in which nucleic acids (NAs) can be amplified under isothermal conditions, with increased specificity, and reaction times of less than 1 hour^26,27^. Modification of the LAMP assay, for reverse transcription amplification (RT-LAMP), has offered a new means by which viral-genome amplification/detection can be utilized for rapid diagnostic testing of COVID-193^24,28,29^. RT-LAMP offers a simplified amplification assay that is imbedded with a reporter system that allows for clear visual interpretation of results. Moreover, with specificity for the SARS-CoV-2 nucleocapsid (N) and spike (S) genes that is comparable to current lab testing methods, RT-LAMP has shown promise as a reliable method for viral detection of SARS-CoV-2^30^. Therefore, the aforementioned benefits of RT-LAMP, coupled with its increased applicability^31,32^ in clinical point-of-care settings, suggest RT-LAMP as a viable option for large-scale rapid diagnostic testing of COVID-19.

Our RT-LAMP assay employs a multiplex amplification approach^33^, utilizing a 6-primer set (3 forward, 3 reverse) to create multiple reverse transcription start sites allowing for accelerated elongation. Specifically, the inner primers (FIP & BIP) bind to viral RNA and complementary DNA, respectively, whereby forward/reverse primers (F3 & B3) allow for multiple-site elongation. Subsequently, complementary sequences at specific positions allow for the DNA product to adopt a hairpin structure whereby the polymerase binds two loop-primers (LF & LB) for amplification of target viral genes^34^. This process requires an RNA-dependent DNA polymerase with strong strand-displacement ability^35^, and tolerance for increased temperatures-typically incubation at 65 °C for 30–40 minutes^36^-which eliminates the need for a thermocycler^34,37,38^ with fluorescence detection. Instead, detection of viral RNA can be achieved by a multitude of different methods, using RT-LAMP; one of which is to use a pH indicator (e.g., phenol red) in a low-buffer reaction. During the amplification, the production of protons, as a result of extensive DNA polymerase activity, subsequently causes a drop in pH, producing a change in solution color from pink to yellow^39^ can be visibly read out in 15–40 min, without a need of any specialized and expensive laboratory equipment.

In this report, we introduce a newly developed rapid diagnostic test for COVID-19 that is independent of conventional testing method requirements and offers increased test throughput. This robust RT-LAMP-based assay allows for rapid on-site testing with minimal set-up requirement. Using saliva as testing specimen, this test provided 94.29% and 98.28% accuracy on detecting positive samples, when compared side-by-side with two FDA EUA approved COVID-19 molecular diagnostic methods, SalivaDirect and Thermo Fisher Scientific TaqPath, respectively. Additionally, this test carries 100% specificity to SARS-CoV-2 virus, with no cross-reactivity detected with common respiratory or other viral and bacterial pathogens. Together with the implementation of customized multiplex primer sets, targeting two distinct regions of the N gene of SARS-CoV-2 genome, and an innovative, pre-loaded sample processing step to specifically address the safety requirements and limited resources available for on-site testing, our new test, SaliVISION, has demonstrated great potential for its use as a reliable on-site testing platform in the continued screening and containment of the spread of COVID-19, especially with the recent increase of more contagious, dangerous variants of SARS-CoV-2 virus.

## Materials and methods

### Clinical sample collection and handling

The biospecimens in this study were obtained from both retrospective and prospective collections. For retrospective collection, this study used leftover nasopharyngeal swabs and saliva samples that were previously tested by the Yale Pathology Labs at the Department of Pathology, Yale School of Medicine for the clinical diagnosis of Covid-19. For prospective collection (on-site study), saliva samples were donated by symptomatic individuals who came to a Yale Health testing site for screening. An informed consent was obtained from all participating subjects through a form of verbal consent and a waiver of a signed consent, as approved by Yale’s Institutional Review Board.

Nasopharyngeal swab (NP) samples were collected and transported in 3 mL of viral transported medium (VTM), according to CDC and FDA guideline. This collection is a part of routine clinical testing offered by the Yale Pathology Labs service. For saliva collection, the specimens were obtained from either passive drool or with a Micro•SAL Saliva Collection Kit (Oasis Diagnostics, Vancouver, WA), according to the manufacturer’s instruction. Saliva samples collected from volunteers at the Yale Health testing sites or the Yale New Haven Hospital were processed on-site within 15 min, while those collected for the Yale Pathology’s Covid-19 Lab were processed 1–2 days after collection.

### Sample lysis with lysis buffer

Upon collection, approximately 500 µl of saliva was aliquoted into a 1.5 mL Eppendorf microcentrifuge tube pre-loaded with 100uL of lysis buffer containing 10 mg/mL of Proteinase K (AmericanBio, Canton, MA) to release viral RNA and 2 M Guanidine hydrochloride (Sigma Aldrich, St. Louis, MO) diluted in sterile PBS. Once collected, saliva samples were lysed for 1 min at 1500 rpm. The lysed saliva samples were subsequently heat inactivated for 5 min at 95 °C. After cooled down, the treated samples were subjected to SaliVISION RT-LAMP and Thermo Fisher Scientific TaqPath COVID-19 RT-PCR assays immediately or stores at − 20 °C until further processing.

### Assessment of Saliva’s pH neutralization with dilution buffer

The neutralization of saliva’s pH was assessed with sodium hydroxide solution at different concentrations. Briefly, negative saliva samples presenting low, or neutral pH were neutralized in different concentrations of sodium hydroxide (750 µM, 1 mM, and 1.5 mM). 1 M NaOH stock solution was prepared by dissolving NaOH pellets in nuclease-free water. To test the detection sensitivity of SARS-CoV-2 in these dilutions, these saliva samples were spiked with 20 copies/µl of synthetic SARS-CoV-2 RNA (Twist Bioscience, San Francisco, CA), in a total of 50 copies per final reaction. The diluted saliva samples were then subjected to the RT-LAMP reaction, described below. After 30 min, the reactions were terminated and transferred to an ice-cold metal block for 30 s before the final color was read and a photograph was taken with a cell phone camera.

### SaliVISION multiplex primer design

SARS-CoV-2 genomes spanning clades G, GH, GR, GV, L, O, S, V from the global science initiative and coronavirus genomic database, GISAID, were randomly collected and aligned with Mega7. In the predominantly expressed N gene, two non-overlapping conservative regions, namely N.1 and N.2, were selected as the testing targets. N.1 primers were designed with NEB LAMP Primer Design Tool, and we used a primer set located in N.2 region from a previous study to do a multiplex availability test with the N.1 primers by using Thermo Fisher Scientific Multiple Primer Analyzer^40^. We used a primer set for RNaseP gene from a previous study as the internal control. A 4 nucleotide PloyA insert was introduced as a linker into all forward and backward inner primers, in order to cooperate with the UDG treatment reaction to decrease the risk of contamination from previous reverse transcripts. All multiplex primer sequences were listed in Table 1. BLASTN Somewhat Similar Alignment method was used to align the primers against the SARS-CoV-2 sequences from GISAID, to make sure there were no multiple mismatches for most of the genomes.

**Table 1:** Multiplex primer sequences used in SaliVISION RT-LAMP assay for SARS-CoV-2 detection.

Primer	Oligo Sequence	Final Concentration (µM)
FIP-N1	TCCCCTACTGCTGCCTGGAGGAAAACAGTCAAGCCTCTTCTCG	1.6
BIP-N1	TCTCCTGCTAGAATGGCTGGCAAAAATCTGTCAAGCAGCAGCAAAG	1.6
F3-N1	GCCAAAAGGCTTCTACGCA	0.2
B3-N1	TTGCTCTCAAGCTGGTTCAA	0.2
LF-N1	GCGACTACGTGATGAGGAA	0.4
LB-N1	GGCGGTGATGCTGCTCTT	0.4
FIP-N2	TGCGGCCAATGTTTGTAATCAGAAAACCAAGGAAATTTTGGGGAC	1.6
BIP-N2	CGCATTGGCATGGAAGTCACAAAATTTGATGGCACCTGTGTAG	1.6
F3-N2	AACACAAGCTTTCGGCAG	0.2
B3-N2	GAAATTTGGATCTTTGTCATCC	0.2
LF-N2	TTCCTTGTCTGATTAGTTC	0.8
LB-N2	ACCTTCGGGAACGTGGTT	0.8
FIP-R	GTGTGACCCTGAAGACTCGGAAAAAGCCACTGACTCGGATC	1.6
BIP-R	CCTCCGTGATATGGCTCTTCGAAAATTTCTTACATGGCTCTGGTC	1.6
F3-R	TTGATGAGCTGGAGCCA	0.2
B3-R	CACCCTCAATGCAGAGTC	0.2
LF-R	ATGTGGATGGCTGAGTTGTT	0.4
LB-R	CATGCTGAGTACTGGACCTC	0.4

### SaliVISION assay with the colorimetric reverse transcription loop-mediated isothermal amplification (RT-LAMP)

Inactivated saliva samples were subjected to one-step reverse transcription loop-mediated isothermal amplification (RT-LAMP), using our customized multiplex primer set that specifically targets two distinct regions on the N gene of SAR-CoV-2 viral genome. The inactivated and pH neutralized saliva samples (pH 7) were processed for RT-LAMP reactions using WarmStart Colorimetric LAMP 2X Master Mix with UDG (New England Biolabs, Ipswich, MA), according to the manufacturer’s protocol. Briefly, the assays were assembled in total reaction volumes of 40 µl, including 2.5 µl of original saliva sample, 20 µl of Warmstart Colorimetric LAMP 2X Master Mix with UDG, 4 µl of 10X customized multiplex primer set, 1 µl of 1 M guadinine hydrochloride, and filled up with RNA-, DNA-free water. The reactions were incubated in the Fisherbrand Isotemp Digital Dry Block Heater (Fisher Scientific, Waltham, MA) set at 65 °C. After 30 min, the reactions were terminated and transferred to an ice-cold metal block for 30 s before the final color was observed and a photograph was taken with a cell phone camera.

### SalivaDirect RT-qPCR

For SARS-CoV-2 detection in saliva samples with the FDA EUA approved SalivaDirect assay, saliva specimens were aliquoted into 96-well plates, in the amount of 50 µl, and subsequently treated with 2.5 µl of 50 mg/mL proteinase K (AmericanBio, Canton, MA). Sample plates were shaken for 1 min, wherein they were then incubated at 95 °C for 5 min for proteinase K inactivation. After that, the RT-qPCR was performed using TaqPath 1-Step RT-qPCR Master Mix (Thermo Fisher Scientific, Waltham, MA) with SalivaDirect primer and probe set in a total volume of 20 µl per reaction, of which 5 µl was for the saliva sample. The samples were run using a Bio-Rad CFX96 Touch qPCR cycler. SalivaDirect’s RT-PCR was used for viral nucleic acid detection with two probes: FAM probes for the presence of N1-gene amplicons, and Cy5 probes for the RNase P housekeeping gene. The threshold for positive samples was set at a Ct value of ≤ 40, in line with the SalivaDirect platform; however, exceptions were made based upon other result characteristics.

### Thermo fisher scientific TaqPath COVID-19 RT-qPCR

200 µl of nasopharyngeal swab specimens collected in viral transport medium were aliquoted into a 96-deep well plate, treated with a premixed solution containing of 265 µl of binding solution, 10 µl of total nucleic acid magnetic beads, 5 µl of 50 mg/mL proteinase K, and 5 µl of MS2 phage internal control (Applied BioSystems, Foster City, CA), and then processed to RNA extraction with the Thermo KingFisher Flex Purification System with 96 Deep Well PCR Magmax (Thermo Fisher Scientific, Waltham, CA), in accordance with manufacturer guidelines. 5 µl of RNA extract diluted in 40 µl of dilution buffer were subjected to qPCR using the TaqPath COVID-19 Combo Kit (Thermo Fisher Scientific, Waltham, CA). The reactions were run with the Applied Biosystems 7500 Fast Dx Real-Time PCR System (Thermo Fisher Scientific, Waltham, CA) to detect three viral probes (N-gene, S-gene, and the ORF1-gene) with the MS2 phage probe serving as an internal control. The threshold for positive samples was kept at a Ct value of ≤ 40, notwithstanding samples with Ct values of ≥ 40, present with extenuating circumstances.

### Statistical analysis

All data were analyzed and graphed with GraphPad Prism 9 software (San Diego, CA). Specificity of the SaliVISION test was calculated as a percentage of the negative samples detected by RT-LAMP that were also negative in either SalivaDirect or TaqPath RT-PCR test. Sensitivity of a given Ct interval was calculated as the percentage of the positive samples detected by RT-LAMP that were also positive in either SalivaDirect or TaqPath RT-PCR test. In both cases, 95% confidence intervals were calculated by interpretating the proportion of counts as binomial rates and then computed using the modified Wald method using GraphPad Prism 9 software.

All methods and experiments in this study were performed in accordance with the guidelines and regulations from Yale Environmental Health and Safety.

## Results

### Assessment of pH neutralization in original saliva samples

**Figure 1:**
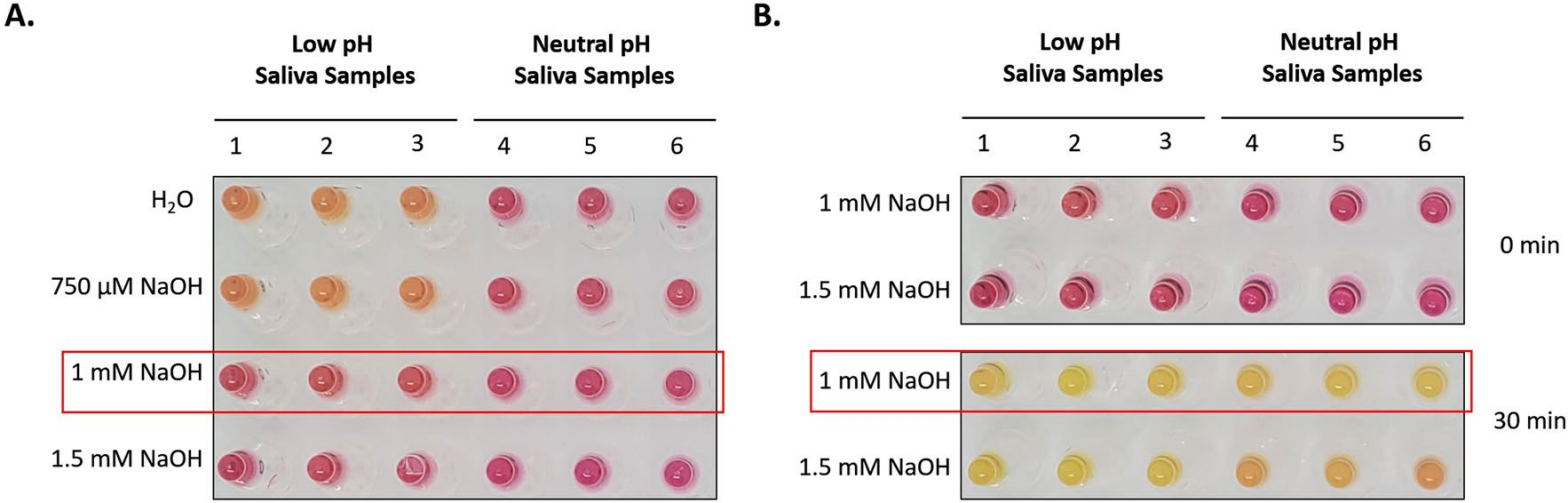
Neutralization of Acidic Saliva Using NaOH Solution. (**A**) Acidic (low pH) and normal (neutral pH) saliva samples were diluted with different concentrations of NaOH solution. The picture was taken immediately after the dilution. The test was performed with 3 independent samples for each group. (**B**) Acidic and normal saliva samples were spiked with 20 copies/µl of synthetic SARS-CoV-2 RNA and then diluted in different concentrations of NaOH solution. The spiked samples were then processed with SaliVISION test at 65 °C for 30 min. The final copy number of synthetic viral RNA was 50 copies per reaction. The red box indicates the optimal concentration of NaOH solution (Dilution Buffer) that meets requirement for both specificity and sensitivity. The images were taken with a phone camera. The tests were repeated 3 times, independently.

The normal range human saliva’s pH is 6.2–7.4, with an average at 6.7. Additionally, approximately 10% of human saliva is acidic^41^. This naturally low pH in primary samples pose a significant problem in the development of a pH-dependent assay, such as the SaliVISION test. To solve this problem, we tested the ability of sodium hydroxide solution (NaOH) in neutralizing the acidity of human saliva. Saliva samples with low pH were diluted with different concentrations of NaOH solution. While the low pH saliva samples diluted in either H_2_O or 750 µM NaOH caused a color change from pink to orange immediately after being added to the WarmStart reaction mix, the color only showed a subtle change when these samples were diluted in 1 mM or 1.5 mM NaOH, indicating that higher concentration of NaOH could neutralize the acidity of saliva and thus maintain the original pH of the WarmStart master mix prior the LAMP amplification (Fig. 1A). Strong base NaOH solution can neutralize the low pH in acidic saliva and enhance the specificity of the test. On the other hand, it may also result in increased alkalinity in saliva with neutral pH, and thus, reduce test sensitivity to these samples. To test this possibility, we performed a SaliVISION assay with spiked SARS-CoV-2 RNA in acidic and neutral saliva diluted with either 1 mM or 1.5 mM NaOH solution. At the NaOH concentration of 1 mM, all acidic and neutral saliva samples showed a strong positive result after 30 min of incubation at 65 °C. The sensitivity of this assay, otherwise, was significantly diminished in neutral saliva samples diluted with 1.5 mM NaOH, when compared to the results of low pH saliva (Fig. 1B). Our data showed that the Dilution Buffer with 1 mM concentration of NaOH is effective to neutralize the natural acidity of saliva sample and stabilize the original pH of the reaction after sample input, without interfering with the sensitivity of the assay. In this test, the assay is designed to tolerate saliva samples with pH ≥ 4.9 while still ensuring stability and reliability for on-site performance.

### Analytical performance of SaliVISION screening and diagnostic test

**Figure 2:**
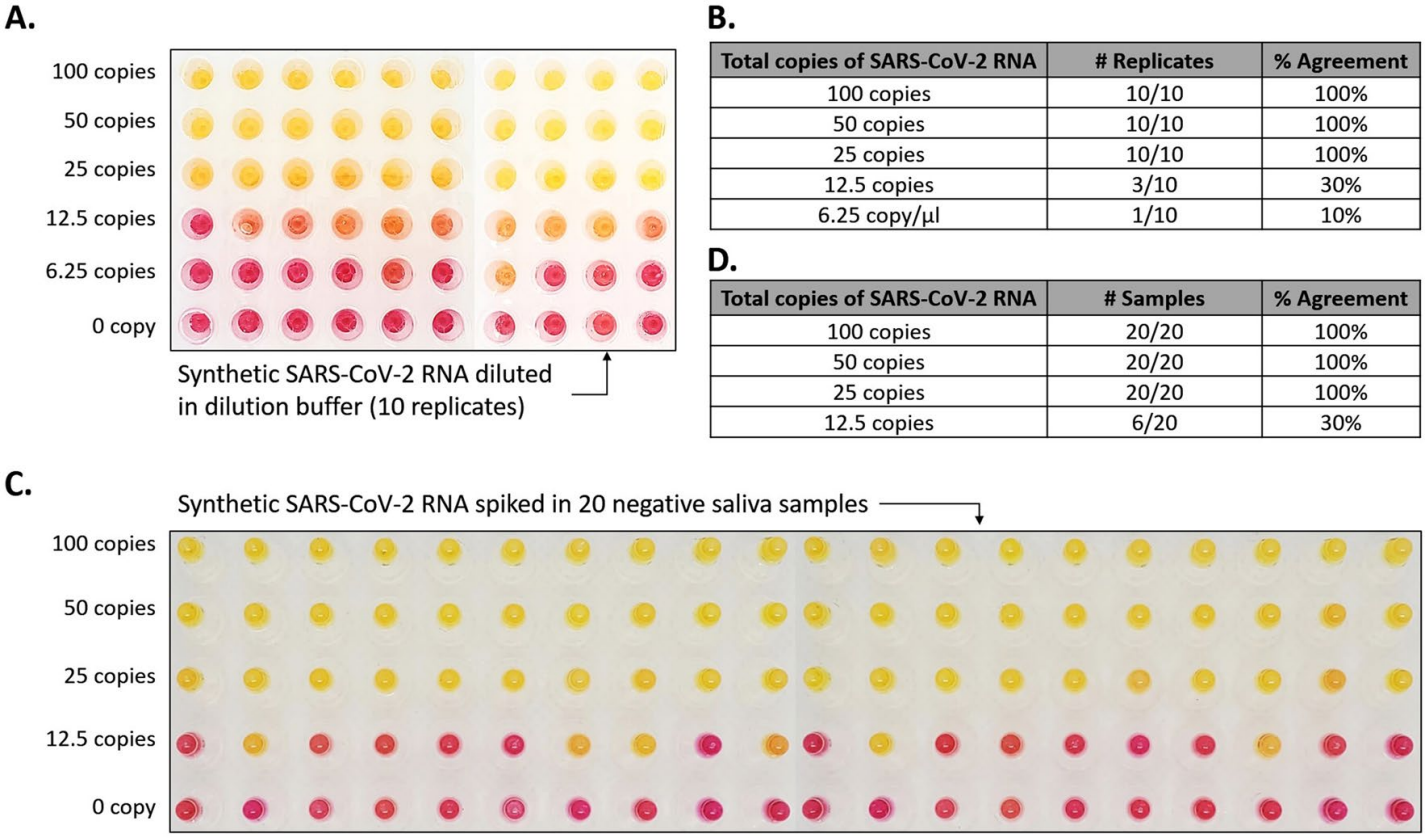
SARS-CoV-2 Detection Limit of SaliVISION Test Using Saliva Samples. Different concentrations of synthetic SARS-CoV-2 RNA was spiked in Dilution Buffer. (**A**) or negative saliva samples (**C**) and subjected to RT-LAMP analysis with SaliVISION test. The reactions were incubated at 65 °C in 30 min. The color change in each sample was captured immediately after the reactions were terminated, using a phone camera. The tables show a summary of positive reactions of 10 independent replicates of viral RNA directly added to Dilution Buffer (**B**) or of 20 negative saliva sampled spiked with viral RNA (**D**). The provided number in these figures and tables represents the actual copy number of viral RNA in each SaliVISION reaction.

The newly developed saliva-based RT-LAMP test’s limit of detection (LoD) was initially assessed with synthetic SARS-CoV-2 RNA (Twist Bioscience, San Francisco, CA) at different concentrations at 10 copies/µl, 5 copies/µl, 2.5 copies/µl, 1.25 copies/µl, and 0.625 copies/µl diluted in dilution buffer. With 10 µl of sample input per 40 µl of total reaction mix, 2.5 copies/µl, or 25 copies total, was determined as the lowest detectable concentration of genomic SAR-CoV-2 RNA, at which 100% of replicates were detected in 10 replicates (Fig. 2 A and B). An additional 10 samples containing only dilution buffer, served as negative controls and to rule out contamination (data not shown).

The LoD of SaliVISION was subsequently verified on 20 negative saliva samples contrived with the same concentrations of synthetic SARS-CoV-2 RNA (Twist Bioscience, San Francisco, CA). These results were in 100% concordance with the results of the aforementioned analytical sensitivity assay, with 2.5 copies/μl being the lowest viral RNA concentration at which 100% of replicates were detected, therefore confirming the LoD of the SaliVISION assay (Fig. 2 C and D).

### Clinical sensitivity and specificity of SaliVISION compared to SalivaDirect testing platform

**Figure 3:**
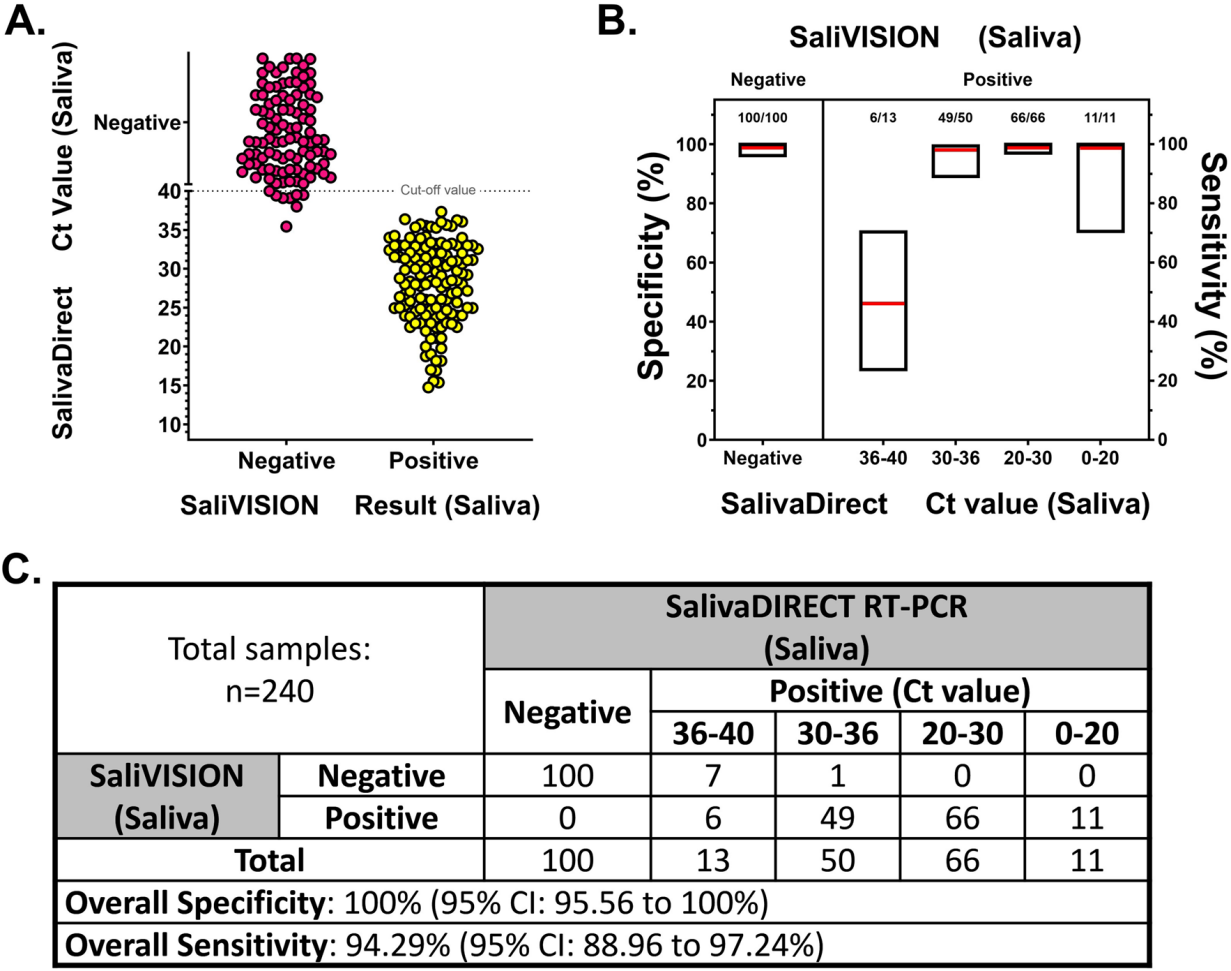
Clinical Validation of SaliVISION, Tested Side-by-Side with SalivaDirect, Using Saliva Samples. Saliva samples being collected for clinical diagnostic test by the Yale Pathology Labs were subjected to SARS-CoV-2 detection using SaliVISION (RT-LAMP) and SalivaDirect (RT-qPCR) methods. In both methods, saliva samples were treated with the lysis buffer and heat-inactivated before being processed. The results of RT-LAMP are compared to relative Ct value determined by quantitative RT-PCR SalivaDirect test. (**A**) The figure represents the distribution of negative (pink) and positive (yellow) samples detected by the SaliVISION test according to their corresponding Ct value detected by the SalivaDirect test (Y axis). The dotted line indicates the cut-off value for SalivaDirect test, in which the samples with Ct value > 40 is considered negative and that with Ct value ≤ 40 is considered positive. (**B**) Specificity (left) and sensitivity (right) of the SaliVISION RT-LAMP assay, derived from data in 3A and C, are shown. For sensitivity, the RT-qPCR positive samples were stratified into four groups, based on the Ct value (x axis). The red lines indicate the value of these proportions. The floating boxes indicate the corresponding 95% confidence intervals computed by Wald’s method. (**C**) The table provides a summary of correlative comparison between the results of SaliVISION and SalivaDirect on the same sample set.

While analytic performance provided a quantitative information on the minimal copy number of the virus that SaliVISION can detect, it is also critical to evaluate its efficacy and accuracy in infected and non-infected clinical samples. Clinical validation consisted of 240 clinical specimens previously collected for SalivaDirect diagnostic test by the Yale Pathology Labs, including 140 positive and 100 negative specimens. The results from the SaliVISION assay were compared to the corresponding Ct values detected by the FDA EUA approved SalivaDirect test for COVID-19 diagnosis. All 100 saliva samples tested negative by SalivaDirect were negative by SaliVISION (100%). Out of 127 positive samples with Ct < 36 detected by SalivaDirect, 126 were positive by SaliVISION (99.21%). Out of 13 positive samples with Ct ≥ 36 detected by SalivaDirect, 6 of them were detected positive by SaliVISION (46.15%). Compared to SalivaDirect RT-PCR testing platform with saliva, the SaliVISION RT-LAMP test has an overall specificity of 100% (Wald’s 95% CI 95.56–100%) and an overall sensitivity of 94.29% (Wald’s 95% CI 88.96–97.24%) (Fig. 3).

### Clinical sensitivity and specificity of SaliVISION compared to thermo fisher scientific TaqPath Covid-19 assay

**Figure 4:**
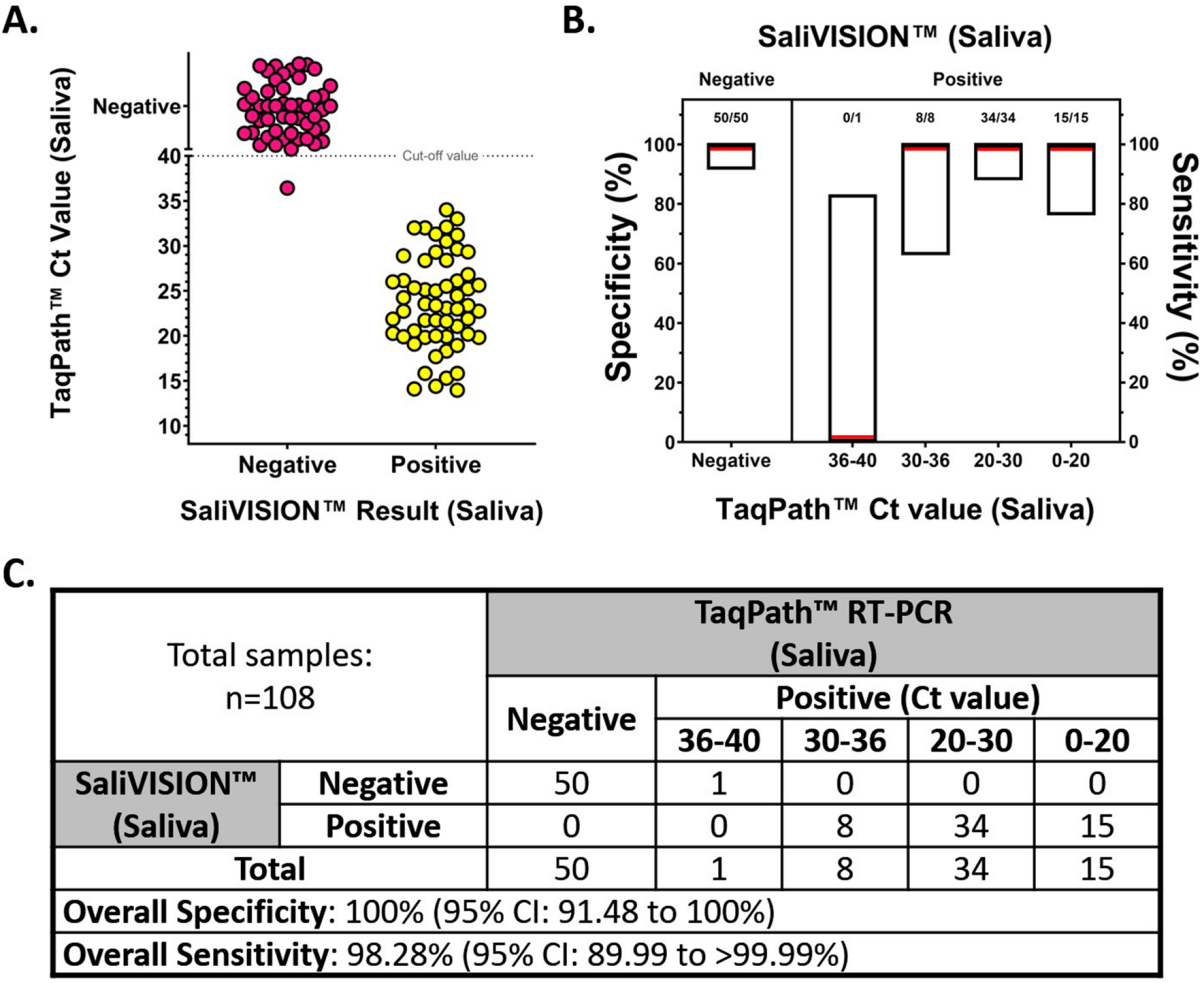
Clinical Validation of SaliVISION, Tested Side-by-Side with TaqPath, Using Saliva Samples. Saliva samples being collected for clinical diagnostic test by the Yale Pathology Labs were subjected to SARS-CoV-2 detection using SaliVISION (RT-LAMP) and Thermo Fisher Scientific TaqPath (RT-qPCR) methods. In both methods, saliva samples were treated with the lysis buffer and heat-inactivated before being processed. The results of RT-LAMP are compared to relative Ct value determined by quantitative TaqPath RT-qPCR test. (**A**) The figure represents the distribution of negative (pink) and positive (yellow) samples detected by the SaliVISION test according to their corresponding Ct value detected by the TaqPath test (Y axis). The dotted line indicates the cut-off value for SalivaDirect test, in which the samples with Ct value > 40 is considered negative and that with Ct value ≤ 40 is considered positive. (**B**) Specificity (left) and sensitivity (right) of the SaliVISION RT-LAMP assay, derived from data in 4A and C, are shown. For sensitivity, the RT-qPCR positive samples were stratified into four groups, based on the Ct value (x axis). The red lines indicate the value of these proportions. The floating boxes indicate the corresponding 95% confidence intervals computed by Wald’s method. (**C**) The table provides a summary of correlative comparison between the results of SaliVISION and TaqPath on the same sample set.

In addition to SalivaDirect, the widely used standard assay for COVID-19 diagnostics, the Thermo Fisher Scientific TaqPath COVID-19 qPCR assay, was performed on different types of samples to elucidate the sensitivity and accuracy of the new SaliVISION test. For saliva samples, our clinical validation consisted of 108 specimens previously collected for COVID-19 diagnostic test by the Yale Pathology Labs, including 58 positive and 50 negative samples. The results from SaliVISION were compared to the corresponding Ct value detected by the Thermo Fisher Scientific TaqPath RT-PCR COVID-19 test. All 50 saliva samples tested negative by TaqPath test were negative by SaliVISION assay (100%). Out of 57 positive samples with Ct < 36 detected by TaqPath, all were positive by SaliVISION (100%). 1 positive sample with Ct ≥ 36 detected by TaqPath was not detected by SaliVISION. Compared to TaqPath RT-PCR testing platform with saliva, the SaliVISION RT-LAMP test has an overall specificity of 100% (Wald’s 95% CI 91.48–100%) and an overall sensitivity of 98.28% (Wald’s 95% CI 89.99 to > 99.99%) (Fig. 4).

### On-site performance of the SaliVISION diagnostic test for COVID-19

**Figure 5:**
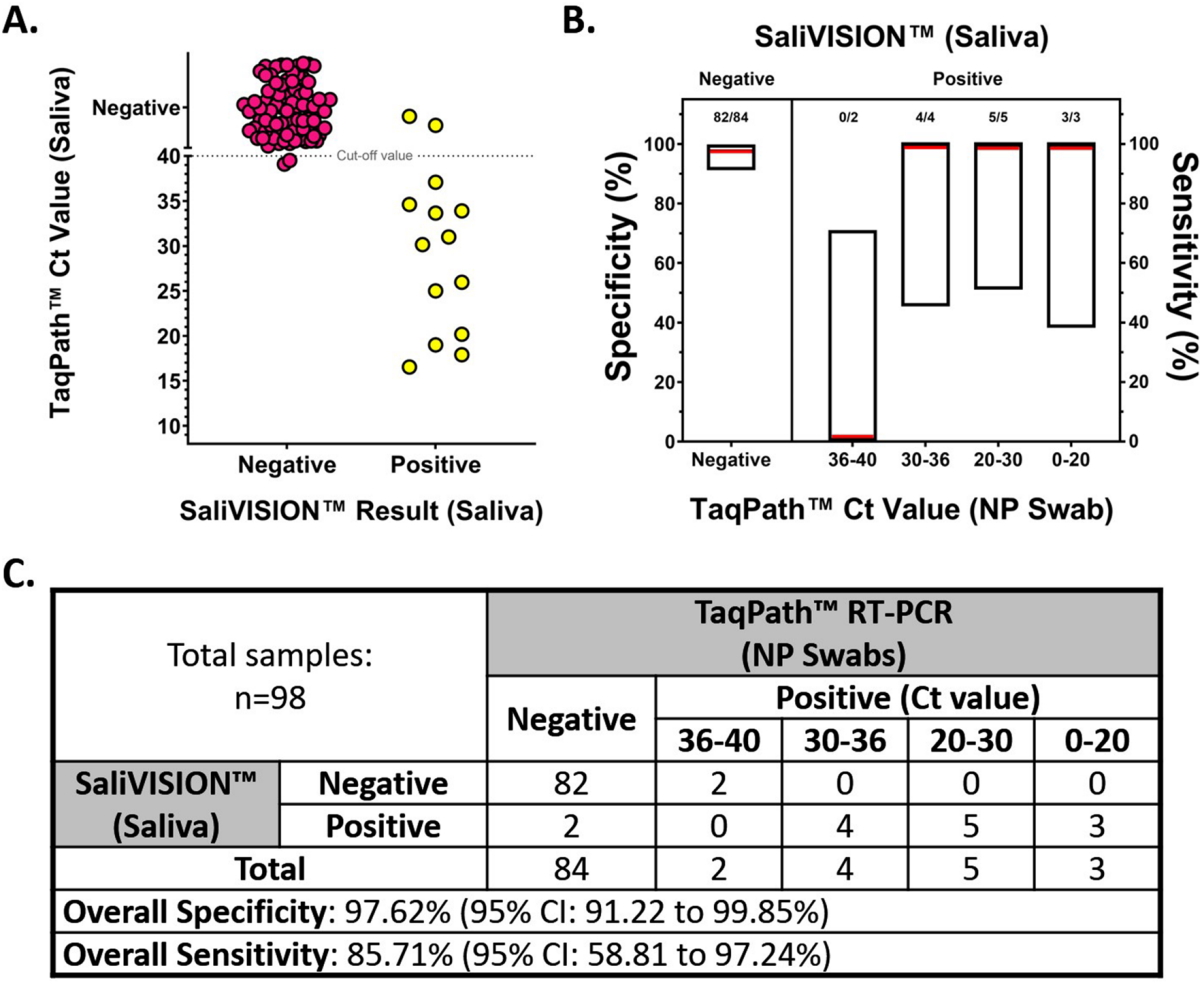
Clinical Validation of SaliVISION, Tested Side-by-Side with TaqPath, Using Saliva and Nasopharyngeal Swab Samples. Pairs of saliva and nasopharyngeal (NP) swab samples being collected from the study participants were subjected to SARS-CoV-2 detection using SaliVISION (RT-LAMP) and Thermo Fisher Scientific TaqPath (RT-qPCR) methods, respectively. For SaliVISION test, saliva samples were treated with the lysis buffer and heat-inactivated before being processed as described previously. This test was performed on-site immediately after the collection. The NP swab samples were processed according to standard protocol as described previously in the following day. The results of RT-LAMP are compared to relative Ct value determined by quantitative RT-PCR TaqPath test. (**A**) The figure represents the distribution of negative (pink) and positive (yellow) samples detected by the SaliVISION test according to their corresponding Ct value detected by the TaqPath test (Y axis). The dotted line indicates the cut-off value for TaqPath test, in which the samples with Ct value > 40 is considered negative and that with Ct value ≤ 40 is considered positive. (**B**) Specificity (left) and sensitivity (right) of the SaliVISION RT-LAMP assay, derived from data in 4A and C, are shown. For sensitivity, the RT-qPCR positive samples were stratified into four groups, based on the Ct value (x axis). The red lines indicate the value of these proportions. The floating boxes indicate the corresponding 95% confidence intervals computed by Wald’s method. (**C**) The table provides a summary of correlative comparison between the results of SaliVISION and TaqPath assays on the same sample set.

On-site performance was conducted in collaboration with the Yale Health Screening Program. In this study, we performed a clinical validation by collecting both saliva and nasopharyngeal swab specimens from 98 symptomatic patients. The result of SaliVISION was compared to the Thermo Fisher Scientific TaqPath COVID-19 RT-qPCR by testing paired saliva and nasopharyngeal samples, on their respective platforms. The SaliVISION assay was performed on-site and processed immediately after sample collection, while NP swab samples were tested by Yale Pathology Labs, the following day. All 84 subjects which tested negative on the TaqPath COVID-19 platform, using nasopharyngeal swab specimens, were negative on the SaliVISION assay, using saliva specimens (100%). Out of the 12 positive NP swab specimens with Ct < 36 detected by the Thermo Fisher Scientific TaqPath assay, all correlated saliva specimens were also positive by SaliVISION (100%). Only 2 positive NP swab specimens with Ct ≥ 36 were detected by the TaqPath assay. Of interest, there were two saliva samples that were detected as positive by SaliVISION test, while their corresponding NP swab samples were detected as negative by the TaqPath RT-PCR test. Compared to TaqPath RT-PCR testing platform with corresponding NP swab samples, the SaliVISION RT-LAMP test has an overall specificity of 97.62% (Wald’s 95% CI 91.22–99.85%) and an overall sensitivity of 85.71% (Wald’s 95% CI 58.81–97.24%) (Fig. 5). This inconsistency in detection may account for the difference in testing materials. In fact, several studies have shown that SARS-CoV-2 can be detected in the saliva of asymptomatic persons, while it was not detected in the corresponding NP swab samples^42,43^. Of interest, a recent finding confirmed SARS-CoV-2 infection in the salivary glands and mucosae and thus, the oral cavity is an important site for SARS-CoV-2 infection^19^.

### Cross-reactivity analysis of SaliVISION assay

Due to the employment of large primer-sets in multiplex PCR assays, the success of a given protocol is largely contingent upon primer design. One such issue is cross-reactivity with other pathogens, given the potential overlap for any one of more than several pairs of primers. Therefore, to ensure our primer design provided adequate specificity for SARS-CoV-2, we performed in silico cross-reactivity analyses of primer sequences. This was done by using BLASTN Somewhat Similar Alignment method to align the 12 primers against the 24 respiratory disease pathogens genomes (Table 2). The average nucleotide identity rate between 314 bps of primer sequences and SARS-CoV-2 is 100%. SARS-CoV has an average nucleotide identity rate of 74.7% with the primer sequences, while in each primer set, at least one primer has an identity rate lower than 25% with SARS-CoV. All other pathogens have no significant overall homologous sequences with the primers, except MERS-CoV, which has a 66% identity rate with one single primer, and Chlamydia pneumonia CWL-029, which has an 84% identity rate with another single primer. Since each primer set needs 6 primers to coordinate for a successful amplification, it is unlikely for these partial homologous primers to cause false positives.

**Table 2:** In silico Cross-reactivity analysis of the SaliVISION multiplex primer set.

Targeted pathogens	GenBank
SARS-CoV-2	NC_045512.2
SARS-CoV	NC_004718.3
MERS-CoV	NC_019843.3
Human Coronavirus 229E	NC_002645.1
Human Coronavirus OC43	NC_006213.1
Human Coronavirus HKU1	NC_006577.2
Human Coronavirus NL63	NC_005831.2
Adenovirus 3	DQ086466.1
Adenovirus 31	DQ149611
Bocavirus	NC_012564.1
Chlamydophila pneumoniae	CWL-029
Enterovirus 68	KP745766.1
Influenza A (H1N1)	NC_026431.1
Influenza A (H3N2)	NC_007366.1
Influenza B	NZ_CP009610.1
Legionella pneumophila	NZ_CP015927.1
Metapneumovirus 8	NC_039199.1
Mycoplasma pneumonia M129	NC_000912.1
Parainfluenza virus 1	NC_003461.1
Parainfluenza virus 2	NC_003443.1
Parainfluenza virus 3	NC_001796.2
Parainfluenza virus 4	NC_021928.1
Rhinovirus 1A	NC_038311
Respiratory syncytial virus	NC_001803.1

Following in silico analysis of the SaliVISION multiplex primer set, we conducted wet testing of our primer set to definitively rule out the possibility of cross-reactivity with our assay. The specificity of SaliVISION multiplex primer set against common respiratory and viral pathogens was tested with purified, intact viral particles, cultured RNA, or bacterial cells commercially available from ZeptoMetrix Corporation (Table 3). 50 μl of each stock was spiked into a pooled negative saliva samples and tested with the SaliVISION assay. No positive signal was detected in any samples, indicating that this assay is specific for detecting SARS-CoV-2, with no cross-reactivity to common respiratory or other viral pathogens, which would generate potential false-positive results (Table 4).

**Table 3:** Commercially available pathogen panels for cross-reactivity wet testing with SaliVISION assay.

Product	Catalog/Lot
NATtrol Respiratory Pathogen Panel-1	NATRPP-1, Lot: 323,985
NATrol Respiratory Validation Panel 3	NATRVP-3, Lot: 323,354
NATtrol Pneumonia Panel—Atypical Bacteria & Viruses	NAPPA-BIO, Lot: 323,680
MERS-CoV-Culture Fluid (Heat Inactivated)	0810575CFHI, Lot: 325,281

**Table 4:** Cross-reactivity wet testing of SaliVISION assay with CDC-recommended common respiratory and viral pathogens.

Organism	# Detection	Organism	# Detection
Adenovirus 3	0/4	RSV B	0/4
Adenovirus 31	0/4	Parainfluenza 1	0/4
Chlamydia pneumoniae	0/4	Parainfluenza 2	0/4
Legionella pneumophila	0/4	Parainfluenza 3	0/4
Mycoplasma pneumoniae	0/4	Parainfluenza 4A	0/4
Mycoplasma pneumoniae M129	0/4	Parainfluenza 4B	0/4
Human metapneumovirus	0/4	Enterovirus	0/4
Metapneumovirus 8	0/4	Rhinovirus	0/4
Parainfluenza 1	0/4	Human bocavirus	0/4
Rhinovirus 1A	0/4	Coronavirus 229E	0/4
RSV A2	0/4	Coronavirus SARS	0/4
Influenza AH3	0/4	Coronavirus OC43	0/4
Influenza A H1	0/4	Coronavirus MERS	0/4
Influenza A/H1N1/2009	0/4	Coronavirus NL63	0/4
Influenza A/H1nN1	0/4	Coronavirus HKU-1	0/4
Influenza A/H3N2	0/4	Respiratory Syncytial A	0/4
Influenza B	0/4	Respiratory Syncytial B	0/4
RSV A	0/4	NATrol Pool 3, 4, 5	0/4

### In Silico analysis for the inclusivity of SaliVISION multiplex primer set

Given the rapid evolution of pervasive, and in some cases, increasingly infectious SARS-CoV-2 variants, the robustness of Covid-19 testing methods is of paramount importance. To account for the potential presence of different SARS-CoV-2 strains, we performed in silico analysis for the inclusivity of our multiplex primer sets. In our assay design, there are two primer sets targeting 2 non-overlapping N gene regions of the SARS-CoV-2 sequence, set N1 and set N2, with each primer set comprised of 6 primers. A total of 200 complete high coverage SARS-CoV-2 sequences spanning clades G, GH, GR, GV, L, O, S, V from GISAID were randomly collected, with specimen collection time from December 2019 to November 2020. BLASTN Somewhat Similar Alignment method was used to align the primers against the SARS-CoV-2 sequences.

BLASTN results showed that the N1 primer set has a primer carrying 1 mismatch for 3% of the strains and another primer carrying 1 mismatch for 2% of the strains. In comparison, the N2 primer set has a primer carrying 1 mismatch for 2% of the strains and another primer carrying 1 mismatch in 1% of the strains. None of the mismatches are at the 3’ end of the primers, and no multiple mismatches were found simultaneously in the same primer set. The multiple mismatches causing false positivity are evaluated with a low possibility, therein revealing the SaliVISION primer sets to be of robust points of detection for the presence of SARS-CoV-2 viral RNA (Table 5).

**Table 5:** In silico inclusivity analysis with SaliVISION multiplex primer set targeting N gene of SARS-CoV-2 virus.

Primer Type	Primer Set N1						Primer Set N2					
	FIP	BIP	F3	B3	LF	LB	FIP	BIP	F3	B3	LF	LB
# of SARS-CoV-2 Strains	200	200	200	200	200	200	200	200	200	200	200	200
0 Mismatch (%)			100	100	100	100	100	100		100		100
1 Mismatch (%)	3	2							2		1	

## Discussion

While public health efforts to curtail the spread of COVID-19 have continuously yielded promising results, continued diagnostic testing constitutes a foremost requirement in the prevention, and early detection of viral infection. Here, we report a new, non-invasive RT-LAMP-based assay for the rapid detection of SARS-CoV-2 in saliva, encompassing novel components for enhanced test accuracy. Following comparative analyses of the SaliVISION assay, in conjunction with both SalivaDirect and Thermo Fisher Scientific TaqPath FDA EUA-approved diagnostic testing platforms, the SaliVISION assay offers 94.29% and 98.28% accuracy, respectively. Moreover, we report 100% specificity for SARS-CoV-2, with no presentable cross-reactivity with other pathogens. Additionally, careful primer design for the targeted N-gene of the SARS-CoV-2 genome, with the inclusion of novel spacer-loop elements, have yielded greater amplification fidelity, and have contributed to the robustness of our test, in spite of a myriad of pervasive SARS variants. Furthermore, the versatility and scalability for which this assay presents allows for increased point-of-care and high-throughput testing capabilities, with a robustness that allows for consistent detection of SARS-CoV-2^44^.

Thus far, molecular diagnostics that have been designed for the diagnosis of COVID-19 have presented an array of challenges which have been further compounded by a myriad of different factors. The newly developed SaliVISION test is not an exception. The primary challenge in this pH-based assay is the original pH of the saliva samples that can significantly impact to the stability of the reporter system and thus affects the specificity of the result. The normal range human saliva’s pH is 6.2–7.4, with an average at 6.7. Additionally, approximately 10% of human saliva is acidic^41^. The pH in these samples could reduce the pH of the WarmStart master mix and cause a color change in the final reaction, despite the absence or presence of the SARS-CoV-2 DNA, leading to a false positive result. To solve this problem, we diluted the inactivated saliva samples with a defined concentration of NaOH to neutralize the (1) natural acidity of saliva sample, and (2) stabilize the original pH of the reaction after sample input, without interfering with the sensitivity of the assay. In addition, a precise dilution factor was optimized to provide an adequate sample input to the final reaction, preventing inhibitory effects due to overwhelming concentrations, thus enhancing both efficiency and efficacy of the assay. In this test, the assay is designed to tolerate saliva sample with pH ≥ 4.9 while still ensuring adequate stability and reliability for on-site performance. In the interest of preventing cross-contamination, we have incorporated Uracil-DNA Glycosylase (UDG) into our assay as a means of preventing amplicon contamination from antecedent reactions^45,46^. Moreover, each reaction strip is completely closed prior to amplification and remains so as visualization of results occurs simultaneously with amplification. Lastly, because the success of RT-LAMP reactions is largely contingent upon successful primer design, several amendments to our primer sets, over the course of test development, were made necessary to maximize test specificity. As it stands, the SaliVISION assay provides 100% specificity for SARS-CoV-2, utilizing a two-primer set for distal locations on the N-gene. The specificity for which this test presents is also, in part, attributed to a novel spacer-loop element which is purposed for increased amplification fidelity for viral SARS-CoV-2 RNA.

Given the projected prevalence of asymptomatic persons carrying COVID-19, designated as asymptomatic spreaders, as well as the increase of new variants with more contagious characteristics, high testing sensitivity in molecular diagnostics for COVID-19 is of paramount importance. Moreover, as some studies have suggested high false-negative rates among RT-PCR diagnostics for COVID-19^47–50^, the importance of high-sensitivity testing is further underscored as the continuation of false-negative diagnoses perpetuate the spread of COVID-19. RT-LAMP has since served as a popular alternative to traditional laboratory diagnostics due to its relative ease-of-use and overall versatility. However, current RT-LAMP tests typically provide a 75%-91% clinical sensitivity^28,51,52^ and offer a limit of detection-in congruence with the reported sensitivity-corresponding to a range of Ct values from ~ 30–33.5^53–55^. We report that the SaliVISION assay has an overall clinical sensitivity of 99.46% for saliva specimens with a Ct value of ≤ 36, as confirmed on both SalivaDirect and TaqPath testing platforms. Moreover, when specifically compared to the TaqPath NP swab testing platform using 98 paired patient samples, collected on site, the SaliVISION assay displayed an overall specificity and sensitivity of 97.62% and 85.71%, respectively; however, among samples with a Ct value of ≤ 36, assay sensitivity was 100%. Although this is not ideal-as the standard convention for ruling out the possibility of COVID-19 infection is confirmed when a Ct value of viral RNA is > 40,-the relative increase in sensitivity for which the SaliVISION assay provides, coupled with its ease-of-use, make it a reliable alternative to traditional laboratory diagnostics, which may be less cost and time efficient.While saliva has been established as a less reliable source of bodily excretion for COVID-19 molecular diagnostics, as opposed to nasopharyngeal excretions, it has become a promising alternative due to less invasive sampling procedures. Further, it has been reported that while saliva typically carries a lighter viral load, the SARS-CoV-2 virus is capable of infecting, and replicating, within cells lining the oral mucosa^19,20^. In addition to infected cells of the lining of oral mucosa, it has also been observed that even in asymptomatic individuals, an acellular fraction of SARS-CoV-2 from infected glands is capable of making de novo virus^19^, therein contributing to infectiousness as well as providing a source of live virus for saliva-based COVID-19 testing. What’s more, the viral longevity in saliva has been reported to be, on average, 18–20 days^10,56,57^, which allows for saliva-based testing to be a valid diagnostic modality for the entire duration of an infected individual’s contagious window. Therefore, saliva has become an appealing source for molecular diagnostic testing; coupled with the associated non-invasive sampling procedures, as well as array of saliva-based rapid testing that currently exists, the use of saliva in COVID-19 testing is rapidly becoming an attractive alternative to other forms of testing.

**Figure 6:**
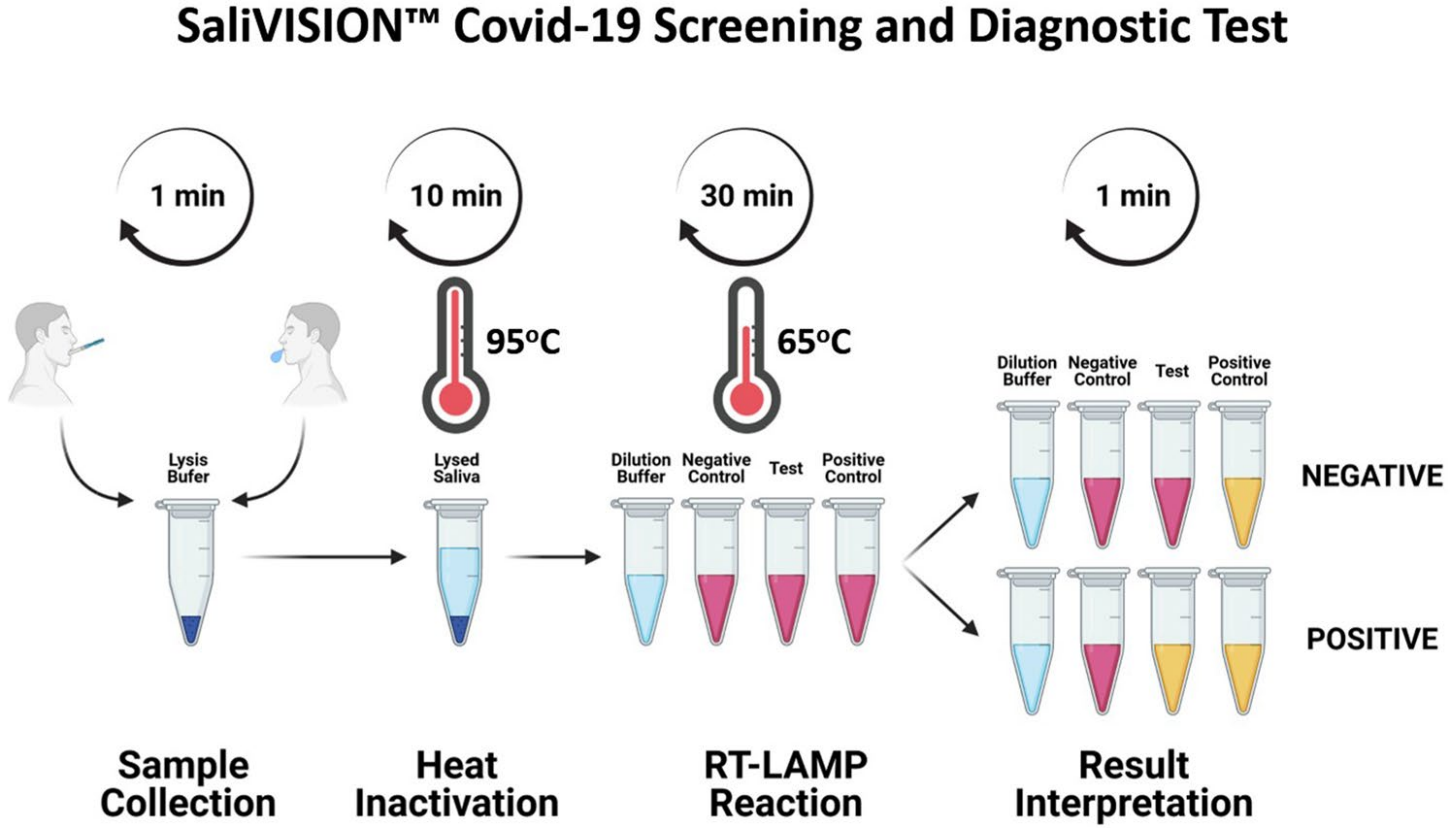
SaliVISION Covid-19 Screening and Diagnostic Approach. (1) The saliva sample self-collected with passive drooling or with MicroSAL Saliva Collection Kit is placed in a Collection Tube pre-loaded with Lysis Buffer; (2) The collected saliva sample in the Collection Tube is lysed and heat-inactivated at 95 °C in 10 min; (3) A fixed volume of inactivated saliva is processed through a serial dilution step in a microtube test strip pre-loaded with Dilution Buffer (#0), negative control (#1), SARS-CoV-2 test with multiplex primers targeting the viral N gene (#2), and internal control with human RNP gene (#3). The RT-PCR reaction will be processed at 65 °C for 30 min; (4) The result is read and interpreted with the cooled reaction mixes after a 30-min incubation with a REPORT CARD.

In the interest of expanding the scope of COVID-19 rapid saliva-based testing, it is imperative to establish a feasible methodology that encompasses stringent safety measures to prevent further spread of the virus. Currently, the body of rapid molecular diagnostics for COVID-19 is primarily comprised of antibody testing; however, such testing is markedly inadequate as the production COVID-19 specific antibodies may take up to several weeks, and thus will not inform an individual of active infection. Other rapid testing modalities include nucleic acid-based testing and antigen testing; the latter has yet to prove reliable in individuals present with low viral loads, while the former, albeit reliable, may still take up to a couple days for results, and is considerably more expensive. Given the foregoing, testing efforts, on all scales, would greatly benefit from an expanded repertoire of readily available rapid testing. Our SaliVISION assay provides enhanced specificity and sensitivity when compared to other modes of rapid diagnostic testing, while proving more time efficient, providing test results within 45 min. In congruence with these benefits, RT-LAMP provides a cheaper alternative testing method due to its minimalistic approach with regards to equipment and reagents. Comparatively, both SalivaDirect and TaqPath testing platforms require a minimum of several hours to process samples, and often take up to 24–48 h for the results to made available. Additionally, the necessity for specialized equipment and instrumentation (e.g. biological safety cabinet, qPCR machine, qPCR consumables/reagents, etc.) can easily triple sample processing costs when compared to the SaliVISION assay. Moreover, we utilize an intuitive, convenient self-sample collection process that allows for increased throughput, while mitigating health-exposure risks (Fig. 6). Further, self-collection accommodation with pre-loaded lysis buffer inside the collection tube allows for specimens to be lysed and inactivated in a closed tube after sample collection, thus circumventing the need for expensive biological safety cabinets and prolonged safety procedures. Therefore, expansion of the RT-LAMP testing platform for the rapid diagnosis of COVID-19 is a promising avenue by which large-scale testing efforts can achieved, more efficiently.

As ongoing efforts to curb the spread of the COVID-19 global pandemic-such as mass vaccination-continue, the demand for readily accessible diagnostic testing remains to be of unequivocal importance, until the efficacy and accessibility of existing vaccines is better established. The early detection, and isolation of pre-symptomatic and asymptomatic individuals with COVID-19 is critical in mitigating the transmission of SARS-CoV-2. With its combined ease-of-use, accuracy, and rapid turn-around time, the SaliVISION assay has the potential to serve as a means of curbing the spread of the COVID-19 pandemic, particularly in stemming asymptomatic transmission. Moreover, when used in conjunction with other easily accessible technologies, such as smartphone-based microfluidic systems of detection, the beneficial impact of the RT-LAMP platform is magnified due to its ease-of-use, analytical robustness, and potential role as a test result database^58^. To this end, the capability to increase testing throughput and accessibility, in addition to another means of monitoring the spread of COVID-19, provide yet another tool in the fight against the COVID-19 pandemic. Therefore, given the versatility of our RT-LAMP assay, we aim to eventually modify this technology to allow for the rapid diagnostic testing of other viral pathogens, for which detection is possible through salivary extracts.
